# Evaluation of the Tuberculosis Infection Care Cascade Among Pregnant Individuals in a Low-Tuberculosis-Burden Setting

**DOI:** 10.1093/ofid/ofae494

**Published:** 2024-08-28

**Authors:** Jeffrey I Campbell, Dorine Lavache, Ariane Garing, Vishakha Sabharwal, Jessica E Haberer, Melanie Dubois, Helen E Jenkins, Meredith B Brooks, Naima T Joseph, Katherine Kissler, C Robert Horsburgh, Karen R Jacobson

**Affiliations:** Section of Pediatric Infectious Diseases, Boston Medical Center, Boston, Massachusetts, USA; Section of Pediatric Infectious Diseases, Boston Medical Center, Boston, Massachusetts, USA; Section of Pediatric Infectious Diseases, Boston Medical Center, Boston, Massachusetts, USA; Section of Pediatric Infectious Diseases, Boston Medical Center, Boston, Massachusetts, USA; Center for Global Health, Massachusetts General Hospital, Boston, Massachusetts, USA; Division of Pediatric Infectious Diseases, Weill Cornell Medical Center, New York, New York, USA; Department of Biostatistics, Boston University School of Public Health, Boston, Massachusetts, USA; Department of Global Health, Boston University School of Public Health, Boston, Massachusetts, USA; Department of Obstetrics and Gynecology, Boston Medical Center, Boston, Massachusetts, USA; College of Nursing, University of Colorado, Aurora, Colorado, USA; Department of Global Health, Boston University School of Public Health, Boston, Massachusetts, USA; Division of Infectious Diseases, Boston Medical Center, Boston, Massachusetts, USA

**Keywords:** care cascade, latent tuberculosis infection, pregnant, referral, tuberculin skin test

## Abstract

In the United States, tuberculosis (TB) screening is recommended for pregnant individuals with TB risk factors. We conducted a retrospective study of perinatal TB infection testing and treatment in a tertiary health system. Of 165 pregnant individuals with positive TB infection tests, only 9% completed treatment within 4.6 years of follow-up.

The American College of Gynecology and the Centers for Disease Control and Prevention recommend routine tuberculosis (TB) screening in pregnant individuals with TB risk factors, such as birth in high-TB-incidence settings, to identify individuals with active TB disease and latent TB infection [1, 2]. While treatment during pregnancy is recommended for active TB, treatment of TB infection is typically deferred until after pregnancy for individuals without risk factors for disease progression, because potential maternal and fetal risk associated with current therapies are thought to outweigh benefits of immediate treatment [3].

The resulting delays between screening and treatment leave pregnant individuals at high risk for loss from TB infection care. A 2016 review [4] identified 3 studies in the United States (US) of pregnant individuals that examined completion of tuberculin skin testing (TST) and treatment with isoniazid. These studies demonstrated high rates of completion of chest radiography following a positive TST, but low rates of treatment completion. Since these reports were published, increased use of interferon-gamma release assays (IGRAs) and rifamycin-based regimens have been associated with increased TB infection care cascade completion in the nonpregnant adult population [5, 6]. In the absence of recent data on TB infection care delivery during pregnancy in the US, we evaluated timing between key events of the care cascade among pregnant individuals to inform strategies to decrease losses from TB infection care.

## METHODS

We conducted a retrospective cohort study of patients tested for TB infection between 1 January 2018 and 31 December 2019 at Boston Medical Center (a tertiary medical center in Boston, Massachusetts) and in affiliated community health centers with linked electronic health records (EHRs). In our setting, pregnant individuals are typically screened for TB infection risk factors [7] during a prenatal visit and tested for TB infection if they have risk factors. TB infection treatment is usually initiated at a dedicated TB clinic.

We identified all patients with a positive TST or IGRA recorded in the EHR whose test was obtained during pregnancy or within 7 days of pregnancy ending. Because pregnancy testing and diagnostic codes were inconsistently available, we relied upon clinical documentation and encounter types to identify pregnant individuals. We were unable to ascertain pregnant individuals with negative tests. From medical records, we abstracted demographic characteristics, pregnancy outcomes, history of TB diagnoses, co-occurring conditions, and timing and completion of each care cascade step. We excluded individuals with documented prior completion of TB infection or disease treatment. For individuals with multiple positive tests during the study timeframe that were obtained in conjunction with pregnancy (n = 6), we only analyzed the first testing episode. We defined follow-up time as the time between the positive test and the last clinical evaluation recorded in the EHR. Because some patients received postpartum care outside our health system, thus making follow-up difficult to ascertain through record review, we excluded patients with <365 days of follow-up in our records to avoid misrepresenting lack of documentation as lack of care access. We performed a sensitivity analysis in which patients were included regardless of follow-up time.

TB infection care cascade steps were defined from prior literature [8]. Patients who received chest imaging from 2 years before through any time after the positive TB infection test were considered to have completed this step. For the remainder of steps, completion consisted of receiving or completing the specified actions at any time after the positive test. We defined referral initiation as an electronic order or clinical documentation of a referral to a TB clinic. We defined referral completion as attending a TB clinic, and treatment initiation as documentation of treatment prescription. We ascertained treatment completion using clinical documentation of clinicians’ assessment.

We used descriptive statistics and cumulative proportions to summarize patient characteristics and completion of care cascade steps. We used Stata version 17 software (StataCorp, College Station, Texas) to perform analyses.

## RESULTS

Of 235 pregnant individuals identified in our record review, 165 met inclusion criteria (Supplementary Figure 1, Supplementary Table 1). The majority (n = 144 [87%]) were born outside of the US, of whom 102 (62%) were born in countries with TB disease incidence of >100/100 000 population and 57 (35%) had immigrated within the preceding 2 years. Human immunodeficiency virus (n = 3 [2%]) and recent known TB disease contact (n = 3 [2%]) were uncommon, while 6 patients (4%) had documented negative TB infection tests within the past 2 years. Most individuals were publicly insured (n = 128 [78%]), non-English speaking (n = 85 [52%]), and lived in census tracts with high social vulnerability, as reflected by a high median Social Vulnerability Index percentile, a composite measure incorporating social factors such as median income, household crowding, and other markers of poverty and access (median, 75th percentile [interquartile range {IQR}, 43rd–92nd percentile]). Most pregnancies ended with term delivery of liveborn infants (n = 135 [82%]). Median clinical follow-up time was 4.6 years (IQR, 4.0–5.2 years).

**Figure 1.**
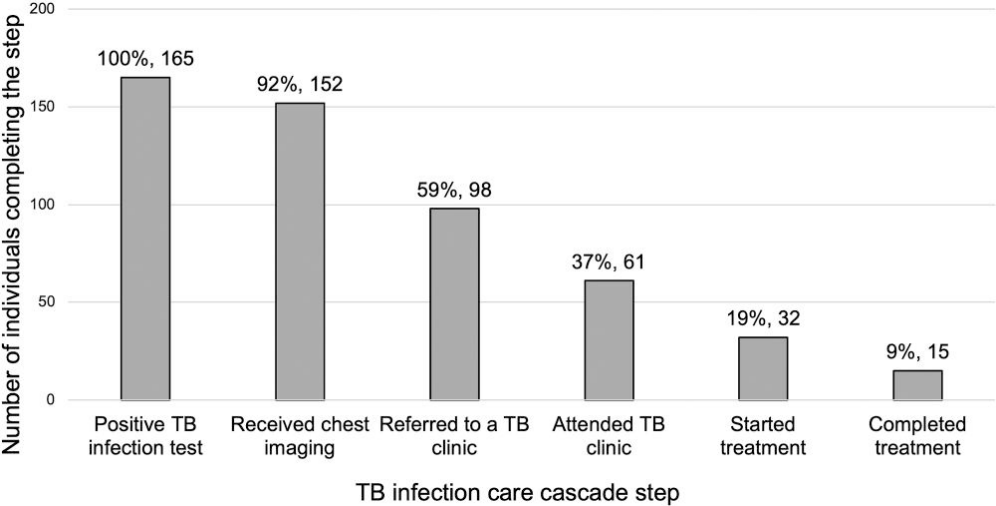
Tuberculosis (TB) infection care cascade in pregnancy.

Figure 1 shows the TB infection care cascade. Most patients received chest imaging (n = 152 [92%]; none with active disease) and referral for TB infection care (n = 98 [59%]). However, only 61 (37%) patients attended TB clinic. Few patients were prescribed (n = 32 [19%]) or completed (n = 15 [9%]) treatment. In a sensitivity analysis that included patients with <365 days of follow-up, losses in each step were similar (Supplementary Figure 2). No patients were prescribed TB infection treatment outside of a TB clinic.

When treatment was deferred due to breastfeeding or pregnancy, subsequent treatment initiation was infrequent. Of the 29 individuals who attended a TB clinic but were not prescribed treatment, treatment was deferred for 27 (93%), of whom 20 (69%) were breastfeeding, 4 (14%) were still or newly pregnant, and 3 (10%) were attempting to conceive. Of these 27 individuals, only 6 (22%) returned to start TB infection treatment.

The time between pregnancy ending and care cascade steps varied (Supplementary Figures 3*A–E* and 4): 23% of individuals were tested between 1 week before to 1 week after delivery, and 28% of individuals who received chest imaging did so in this timeframe. Forty-six percent of individuals who ultimately attended clinic and 25% of individuals who started treatment did so within 1 year of delivery. No patients were prescribed therapy during pregnancy.

Time-to-event analysis showed that the pace of referrals declined after the delivery admission. Supplementary Figure 5*A–D* shows a wide range in timing to evaluation and treatment after the positive test. One patient (0.7%) who never took TB infection treatment was diagnosed with extrapulmonary TB disease 4.3 years after a positive TB infection test in pregnancy.

## DISCUSSION

In this cohort of pregnant individuals with TB infection, most of whom were born in high-TB-incidence countries and lived in neighborhoods with high social vulnerability, we identified high rates of noncompletion of TB infection care cascade steps. Attrition rates from the care cascade in our study were similar to prior analyses of TB infection care among pregnant individuals in the US [4] but lower than analyses among US adults and children [9, 10] and lower than a recent analysis among pregnant individuals in Sweden [11]. Our findings add evidence that pregnant individuals with TB infection in the US are a group at high risk for loss from TB infection care and merit unique solutions to mitigate these losses.

In our cohort, patients experienced delays in ruling out TB disease with chest imaging. Approximately 10% did not complete chest imaging until >1 year after delivery, and 8% never obtained chest imaging. US-based recommendations endorse obtaining chest X-rays for pregnant individuals with positive TB infection tests at time of testing if during the second trimester or later, or during the first trimester for high-risk individuals [12]. Our results highlight gaps in implementation of these recommendations and indicate a need for systems and education to facilitate prompt evaluation for TB disease following a positive TB infection test in pregnancy.

Numerically, most losses from the care cascade occurred during the referral process. The finding that the tempo of referrals declined after the delivery hospitalization suggests that there are missed opportunities to leverage postpartum care for TB infection care engagement. Insurance churn, transitions in care from obstetricians to primary care physicians, and the change in focus from maternal to infant care may also disrupt the referral process. Encouraging clinicians to refer patients early during pregnancy, empowering primary care physicians to treat TB infection, and enabling pediatricians to facilitate care referrals for parents might improve linkage to and initiation of TB infection treatment.

We found that treatment deferral until after pregnancy and breastfeeding often led to permanent loss to follow-up. Data are mixed about the safety of treating TB infection during pregnancy [3, 13]. In our cohort, no individuals received TB infection treatment during pregnancy, likely reflecting patients' and clinicians' aversion to potential risks. Expert opinion also differs about whether TB infection treatment should be deferred for several months postpartum [12, 14]. Treatment with either isoniazid or rifampin is not contraindicated during breastfeeding, but clinicians may be reluctant to initiate therapy due to concerns about breastmilk supply, infant tolerance, milk discoloration, and drug transfer to breast milk. Engaging in shared decision-making about treatment in the immediate postpartum period, including during breastfeeding, may mitigate losses from the care cascade that occur when treatment is deferred postpartum.

Our study has limitations. First, we were unable to ascertain all pregnant individuals who were at risk for and tested for TB infection in our setting, precluding analysis of gaps in testing for TB infection. Second, all clinical data were obtained from the EHR; we were unable to access pregnancy or TB outcome data for patients who transferred to care outside of our system. Third, this was a retrospective study, conducted partly during the COVID-19 pandemic, in a single healthcare system, limiting generalizability.

In conclusion, only 9% of pregnant individuals with a positive TB infection test completed TB infection care within a median of 4.6 years of clinical follow-up. Our findings indicate that strategies that strengthen referral and retention in care are necessary in the pre- or immediate postpartum periods. Because pregnant individuals with TB infection in the US often face social and structural barriers to healthcare, culturally tailored interventions to support and improve cascade completion are needed for this population.

## Supplementary Data

Supplementary materials are available at *Open Forum Infectious Diseases* online. Consisting of data provided by the authors to benefit the reader, the posted materials are not copyedited and are the sole responsibility of the authors, so questions or comments should be addressed to the corresponding author.

## Supplementary Material

ofae494_Supplementary_Data

## Notes

***Acknowledgments.*** We thank the patients, clinicians, and staff of Boston Medical Center and affiliated community health centers.

***Participant consent.*** Patient consent was waived due to research involving only use of retrospective electronic health record data. This study was approved by the Boston University Medical Campus Institutional Review Board.

***Financial support.*** This work was supported by the Joel and Barbara Alpert Endowment for Children of the City, the Boston Medical Center Pediatrics Center for the Urban Child and Healthy Family, and the Boston-Providence Center for AIDS Research. M. B. B. is supported by award number K01AI151083 from the National Institute of Allergy and Infectious Diseases of the National Institutes of Health.
