# Supplementary material for: Evaluation of the Tuberculosis Infection Care Cascade Among Pregnant Individuals in a Low-Tuberculosis-Burden Setting
Source: Open Forum Infect Dis. 2024 Aug 28;11(9):ofae494. doi: 10.1093/ofid/ofae494 (PMC11376066; doi:10.1093/ofid/ofae494)
Supplement: ofae494_Supplementary_Data [file ofae494_supplementary_data.zip › Supplementary_Legends.docx]

**Supplemental figure 1.** Consort diagram.

**Supplemental figure 2.** TB infection care cascade in pregnancy, including individuals with <365 days of clinical follow-up.

**Supplemental figure 3.** Time of care cascade steps in relationship to pregnancy end date. **(A)** Timing of testing; **(B)** timing of chest imaging (data missing for 6 individuals); **(C)** timing of TB clinic referral (data missing for 2 individuals); **(D)** timing of TB clinic attendance; **(E)** timing of treatment prescription**.**

**Supplemental figure 4.** Comparative times to cascade steps. Box plots show median and interquartile ranges, and whiskers show the 90%ile range.

**Supplemental figure 5.** Time of care cascade steps in relationship to testing. **(A)** Timing of chest imaging (data missing for 6 individuals); **(B)** timing of TB clinic referral (data missing for 2 individuals); **(C)** timing of TB clinic attendance; **(D)** timing of treatment prescription**.**

| **Characteristics** | **N = 165** |
| --- | --- |
| Age, median (IQR) | 33 (30-38) |
| Insurance |  |
| Public | 129 (78%) |
| Private | 23 (14%) |
| Other | 2 (1%) |
| Unknown | 11 (7%) |
| Language |  |
| English | 80 (48%) |
| Spanish | 12 (7%) |
| Haitian Creole | 52 (32%) |
| Other | 21 (13%) |
| Born outside of US | 144 (87%) |
| Birth country TB incidence **≤**100/100,000 | 63 (38%) |
| Birth country TB incidence >100/100,000 | 102 (62%) |
| Time since immigration |  |
| 0-<2 years | 57 (35%) |
| 2-<5 years | 28 (17%) |
| 5+ years | 35 (21%) |
| Unknown | 45 (27%) |
| Social Vulnerability Index census tract rank, median (IQR) | 75%ile (43%ile, 92%ile) |
| Comorbidities |  |
| HIV | 3 (2%) |
| Type 2 diabetes | 5 (3%) |
| Rheumatologic disease (including IBD) | 1 (1%) |
| Known TB contact in 2 years prior to TB testing | 3 (2%) |
| Negative tuberculosis test in 2 years prior to TB testing | 6 (4%) |
| Gestations, median (IQR) | 2 (2-4) |
| Pregnancy outcome |  |
| Liveborn term infant | 135 (82%) |
| Liveborn preterm infant (<37 weeks) | 12 (7%) |
| Spontaneous abortion | 14 (9%) |
| Unknown | 4 (2%) |
| Days from TB test to pregnancy end, median (range) | 109 (253, -5) |
| Years from TB test to last clinical record | 4.6 (4.0, 5.2) |
| Testing modality |  |
| TST | 23 (14%) |
| IGRA | 142 (86%) |
| Treatment regimen |  |
| 9H | 20 (63%) |
| 4R | 10 (31%) |
| 3HP | 1 (3%) |
| 4RH (for class IV TB) | 1 (3%) |

**TABLES**

**Supplemental table 1.** Characteristics of pregnant individuals with positive TB infection tests included in the primary analysis.

**Abbreviations:** IQR – interquartile range; IBD – inflammatory bowel disease; TST – tuberculin skin test; IGRA – interferon gamma release assay; 9H – 9 months of isoniazid; 4R – 4 months of rifampin; 3HP – 3 months of isoniazid plus rifapentine; 4RH – 4 months of rifampin plus isoniazid.
