# Supplementary figures and images for: Evaluation of the Tuberculosis Infection Care Cascade Among Pregnant Individuals in a Low-Tuberculosis-Burden Setting
Source: Open Forum Infect Dis. 2024 Aug 28;11(9):ofae494. doi: 10.1093/ofid/ofae494 (PMC11376066; doi:10.1093/ofid/ofae494)

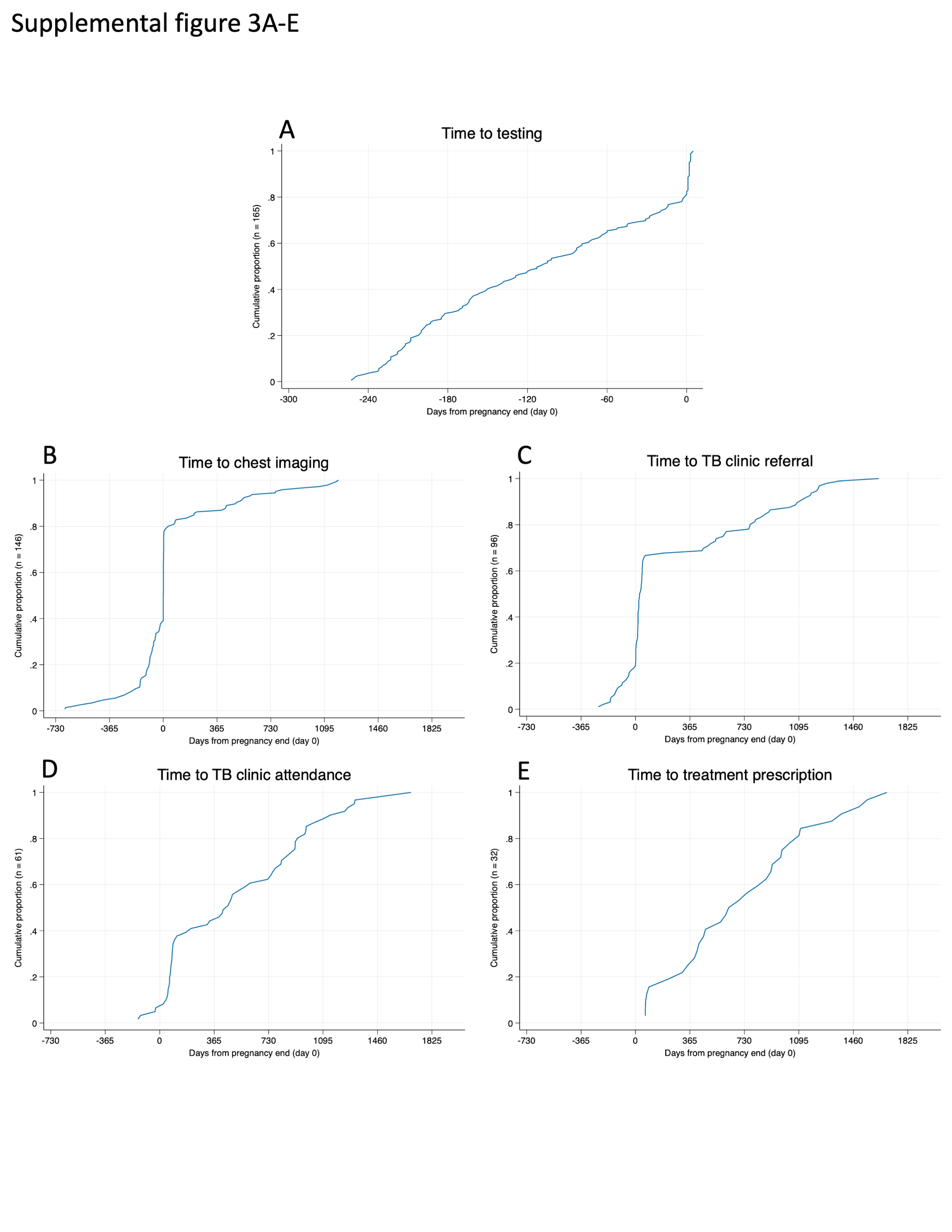

Supplement: ofae494_Supplementary_Data [file ofae494_supplementary_data.zip › Supplemental figure 3.tiff]

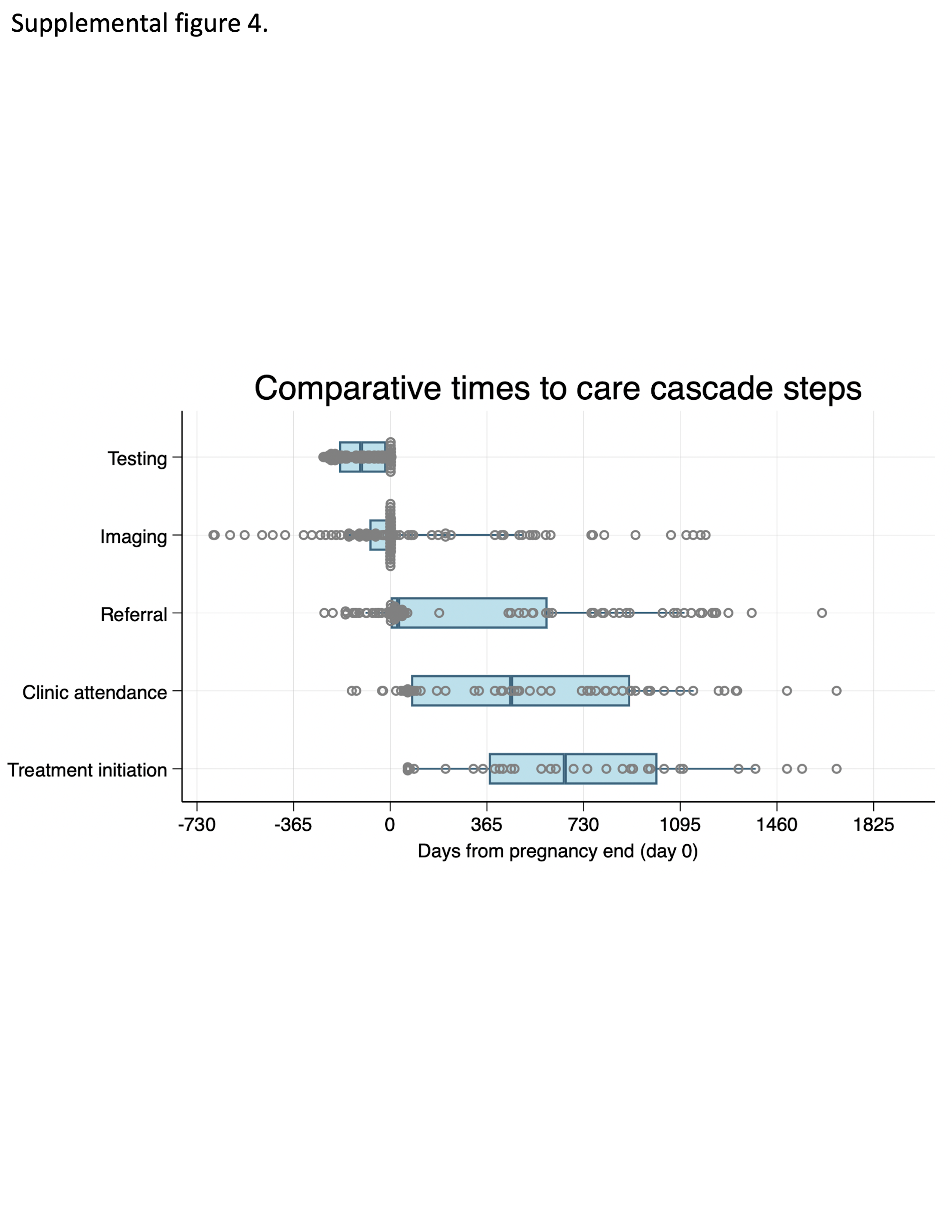

Supplement: ofae494_Supplementary_Data [file ofae494_supplementary_data.zip › Supplemental figure 4.tiff]

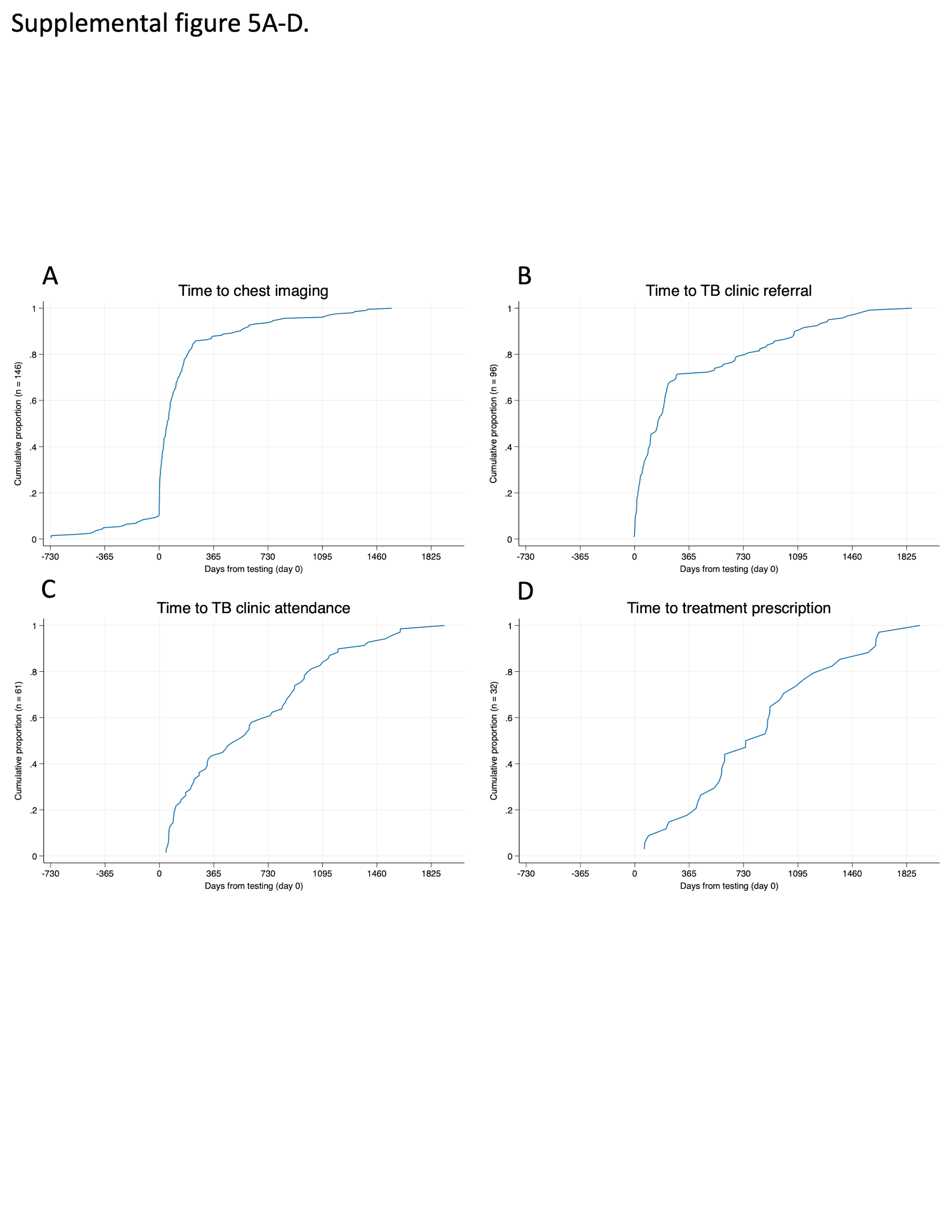

Supplement: ofae494_Supplementary_Data [file ofae494_supplementary_data.zip › Supplemental figure 5.tiff]

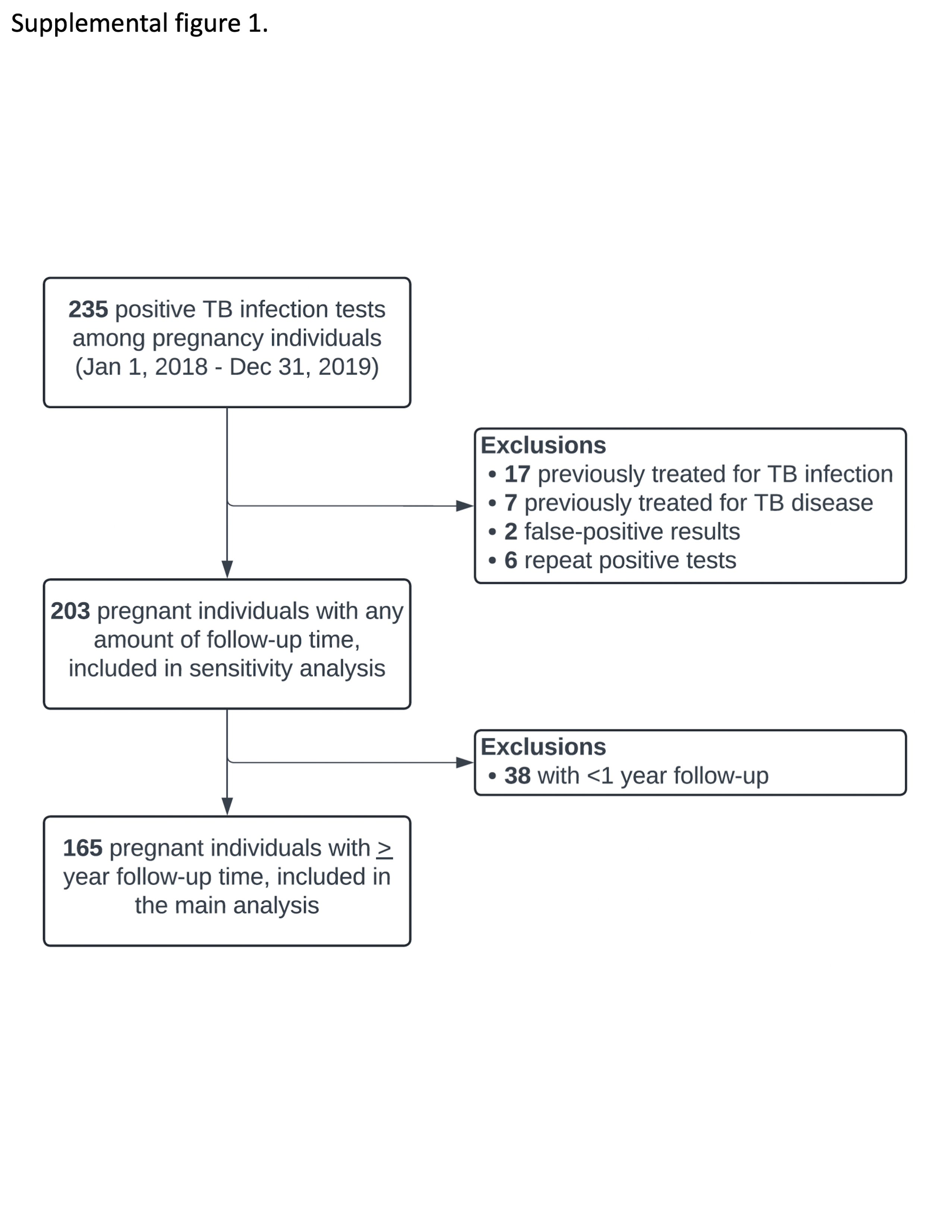

Supplement: ofae494_Supplementary_Data [file ofae494_supplementary_data.zip › Supplemental figure 1.tiff]

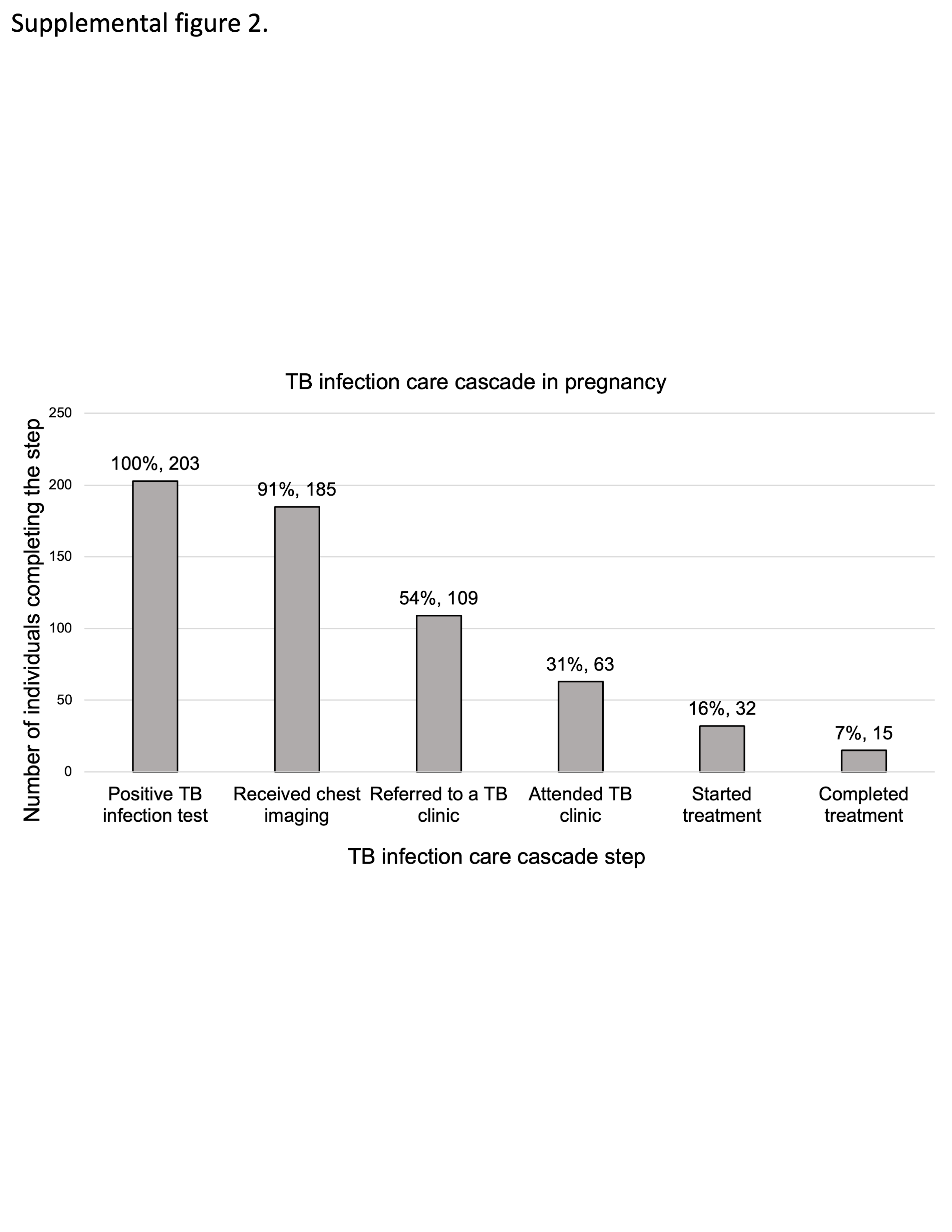

Supplement: ofae494_Supplementary_Data [file ofae494_supplementary_data.zip › Supplemental figure 2.tiff]
